# Polyketide synthase gene clusters in the ericoid mycorrhizal fungus *Oidiodendron maius*: genome-wide prediction, expression profiling, and functional characterization of knockout mutants

**DOI:** 10.3389/fpls.2026.1924430

**Published:** 2026-09-16

**Authors:** Simone Belmondo, Stefania Daghino, Shingo Miyauchi, Marco Chiapello, Ekaterina Shelest, Miriana Bortolot, Annegret Kohler, Christophe Jacob, Silvia Perotto, Elena Martino

**Affiliations:** 1 Department of Life Sciences and Systems Biology, University of Turin, Turin, Italy; 2 Institute for Sustainable Plant Protection, National Research Council of Italy (CNR), Turin, Italy; 3 Evolutionary and Synthetic Biology Unit, Okinawa Institute of Science and Technology, Onna, Okinawa, Japan; 4 Centre for Enzyme Innovation, University of Portsmouth, Portsmouth, Hampshire, United Kingdom; 5 Université de Lorraine, French National Research Institute for Agriculture, Food and Environment (INRAE), Unité mixte de recherche (Joint research unit) (UMR) Interactions Arbres/Microorganismes, Centre INRAE Grand Est Nancy, Champenoux, France; 6 UMR, Ingénierie Moléculaire, Cellulaire et Physiopathologie (IMoPA), French National Centre for Scientific Research (CNRS)-Université de Lorraine, Biopôle de l’Université de Lorraine, Vandœuvre-lès-Nancy Cedex, France

**Keywords:** ericoid mycorrhiza, fungal antagonistic interactions, *Oidiodendron maius*, polyketide biosynthesis, secondary metabolites

## Abstract

**Introduction:**

Fungal polyketides constitute a structurally diverse class of secondary metabolites involved in ecological adaptation, stress responses, antagonistic interactions, and host-associated processes. However, their roles in mycorrhizal fungi remain poorly understood. In this study, we investigated the repertoire and potential functions of polyketide synthases (PKSs) in the ericoid mycorrhizal fungus *Oidiodendron maius*.

**Methods and Results:**

Genome-wide analyses identified 45 PKS-coding genes and 59 predicted biosynthetic gene clusters associated with secondary metabolite production. Transcriptomic analyses revealed that several PKS genes were differentially regulated during symbiosis with *Vaccinium myrtillus* and under cadmium exposure. Among these, the gene *OmPKS197601* was strongly upregulated under both conditions and was selected for functional characterization through Agrobacterium tumefaciens-mediated gene disruption. Three independent OmPKS197601 knockout mutants were generated and evaluated for their ability to establish mycorrhizal symbiosis and tolerate cadmium and zinc stress. Knock-out mutants were also tested for their ability to antagonize the growth of selected fungal species. No significant differences were observed between the wild-type strain and the three *OmPKS197601* knock-out mutants in either mycorrhization efficiency or metal tolerance. In contrast, dual-culture assays with the M2 knock-out mutant demonstrated reduced antagonistic activity against both plant pathogenic and saprotrophic fungi, including *Heterobasidion annosum, Pythium* sp., *Kuehneromyces mutabilis*, and *Stereum hirsutum*.

**Conclusions:**

Overall, these findings reveal the extensive genetic potential of *O. maius* for polyketide biosynthesis and suggest that regulated expression of PKS genes may contribute to the ecological fitness and biocontrol potential of ericoid mycorrhizal fungi.

## Introduction

The production of natural metabolites by living organisms is of major ecological and biotechnological importance owing to their remarkable structural diversity and broad spectrum of biological activities (Newman and Cragg, 2007; Bérdy, 2015). Fungi represent a particularly rich source of secondary metabolites (SMs), many of which are exploited as pharmaceuticals, agrochemicals, and industrial compounds, while others are recognized as important food contaminants (Greco et al., 2019). Among fungal secondary metabolites, polyketides constitute one of the largest and most structurally diverse classes of natural products.

Polyketides are synthesized by polyketide synthases (PKSs), which are multi-domain enzymes with highly divergent architectures capable of generating structurally complex molecules. While some polyketides can be harmful, such as mycotoxins contaminating food supplies, many others possess considerable therapeutic value, including antibiotics, anticancer compounds, and immunosuppressive agents (Keller, 2019). Although polyketides are produced by a broad range of organisms, fungal polyketides are among the most abundant and structurally diverse. The secretion of specialized metabolites is essential for fungal survival in competitive environments and play important roles in fungal–plant interactions (Calvo and Cary, 2015). Indeed, several ecological and evolutionary functions have been attributed to fungal polyketides (Keller, 2019; Stroe et al., 2024): they can act as toxin precursors, pigments involved in virulence or resistance to abiotic stresses, antibiotics, and signaling molecules (Eisenman and Casadevall, 2012), and they are also implicated in fungal growth, sexual development, and sporulation processes (Gaffoor et al., 2005; Fatema et al., 2018).

PKSs can be divided into several classes according to their architecture and biosynthetic mechanisms. Among the array of biosynthetic enzymes, fungi predominantly utilize monomodular iterative type I PKS for the biosynthesis of polyketides. These enzymes are composed of multiple domains, each responsible for specific catalytic activities, including the elongation of the polyketide chain and various modifications to the growing molecule (Staunton and Weissman, 2001). Based on domain composition of the enzyme complexes, fungal PKSs are commonly classified into non-reducing (NR), partially reducing (PR), and highly reducing (HR) PKSs (Mattern et al., 2015). As a general rule, NR-PKS and PR-PKS synthesize aromatic polyketides with unmodified ketone groups, while HR-PKS typically produce aliphatic compounds (Gaffoor et al., 2005; Chooi and Tang, 2012).

In addition, fungi frequently possess hybrid PKS–NRPS (non-ribosomal peptide synthetase) enzymes combining polyketide synthase and non-ribosomal peptide synthetase modules, thereby generating structurally complex metabolites with diverse biological activities, including antimicrobial, anticancer, and immunosuppressive properties (Gressler et al., 2021).

To date, extensive research has addressed the role of PKSs in pathogenic interactions and biological control processes (Böhnert et al., 2004; Yao et al., 2016). In the rice pathogen *Magnaporthe oryzae*, for example, the PKS encoding avirulence gene ACE1 is specifically upregulated during fungal penetration of plant tissues (Böhnert et al., 2004). Similarly, metabolites produced by the mycoparasitic fungus *Trichoderma harzianum* are involved in antagonistic interactions with plant pathogens, and two PKSs encoding genes are differently regulated in response to competing fungi (Yao et al., 2016). In contrast, comparatively little information is available concerning the functions of PKSs in fungi that form mutualistic symbioses, namely lichen-forming (Bertrand and Sorensen, 2018), ectomycorrhizal (Löhr et al., 2023) and arbuscular mycorrhizal (Venice et al., 2020) fungi. Recent genomic and phylogenetic studies suggest that PKSs may play important roles in mycorrhizal fungi by contributing to chemical signaling, microbial competition, and ecological adaptation within the rhizosphere (Venice et al., 2020, 2021).

Over 90% of plants establish symbiotic associations with mycorrhizal fungi. Mycorrhizal interactions are found in almost all ecosystems and play fundamental roles in plant nutrition, ecosystem functioning, and biogeochemical cycles (Van der Heijden et al., 2015). Ericoid mycorrhiza is an endosymbiosis that involves mainly ascomycetous fungi and the fine roots of Ericaceae plants (Perotto et al., 2012). Ericaceous plants dominate in heathlands, but they are also found as understory forest vegetation in temperate and boreal ecosystems, as well as in Mediterranean climates. They typically inhabit acidic, nutrient-poor soils frequently characterized by harsh environmental conditions and elevated concentrations of heavy metals. The ecological success of Ericaceae species in these stressful habitats has been largely attributed to the physiological and metabolic capabilities of their fungal symbionts (Cairney and Meharg, 2003). The fungus studied in this work is a widespread ericoid mycorrhizal symbiont belonging to the species *Oidiodendron maius* (class Leotiomycetes), isolated from roots but also peat and decomposing organic matter in temperate ecosystems, including bogs, heathlands, and forest soils (Rice and Currah, 2002). *O. maius* represents an excellent experimental system because it can be cultivated *in vitro*, can be genetically transformed and used in controlled mycorrhizal synthesis assays (Martino et al., 2007). The isolate used in this work, *O. maius* Zn, displays considerable tolerance to heavy metals and represents an established model for investigating mechanisms of cadmium and zinc tolerance in ericoid mycorrhizal fungi (Daghino et al., 2016; Abbà et al., 2009; Di Vietro et al., 2014; Casarrubia et al., 2018). Its genome has been sequenced (Kohler et al., 2015) and transcriptomic datasets generated under symbiotic and stress conditions are publicly available (Martino et al., 2018).

A comparative genomic study involving 60 fungal species with different taxonomic affiliations and ecological strategies revealed that *O. maius* Zn possesses the highest number of PKS-encoding genes among the fungi analyzed (Martino et al., 2018). However, the possible contribution of secondary metabolites to plant-fungal interactions as well as metal tolerance in *O. maius* Zn has not yet been investigated. Aim of this work was therefore to analyse the PKS encoding genes of *O. maius* Zn (Martino et al., 2018), analyse their expression in symbiosis and investigate potential functions of specific PKS-derived metabolites by gene knockout. To reach these aims we: (1) characterized PKS domains and reconstructed the phylogeny of *O. maius* PKS genes; (2) performed a genome-wide scan and *in silico* prediction of biosynthetic gene clusters (BGCs) containing PKS genes and potentially involved in secondary metabolite production in *O. maius*; (3) analyzed the expression profiles of individual PKS genes and BGCs during symbiosis and cadmium exposure; (4) identified candidate genes regulated under these conditions to generate knockout mutants through *Agrobacterium tumefaciens*-mediated transformation; and (5) functionally characterized the resulting mutants to investigate the potential roles of PKS-derived metabolites in symbiosis, metal tolerance, and antagonistic interactions.

## Materials and methods

### Fungal strain and growth conditions

*Oidiodendron maius* strain Zn (hereafter *O. maius*) was isolated from the roots of *Vaccinium myrtillus* growing in the Niepolomice Forest (Poland) and first described by Martino et al. (2000). This *O. maius* strain is deposited at the Mycotheca Universitatis Taurinensis collection (MUT1381; University of Turin, Italy) and at the American Type Culture Collection (ATCC MYA4765; Manassas, VA, US), and was maintained on Czapek Dox solid medium (NaNO_3_2 gL^−1^, KCl 0.5 gL^−1^, glycerol phosphate∗H_2_O 0.5 gL^−1^, K_2_HPO_4_ 0.35 gL^−1^, FeSO_4_ 0.01 gL^−1^, sucrose 30 gL^−1^, agar 10 gL^−1^, adjusted to pH 6).

### Phylogenetic and bioinformatic analysis

#### Prediction and expression profiles of biosynthetic gene clusters (BGCs) of *O. maius*

Biosynthetic gene clusters (BGCs) of *O. maius* coding for potential secondary metabolites were predicted by using antiSMASH 4.1 fungal version (Medema et al., 2011). Calculation was performed on the computing cluster at INRAE, Grand-Est Centre, Nancy, France.

Other features were also predicted in the *O. maius* genome to provide a global overview. Secreted proteins were predicted according to Pellegrin et al. (2015), CAZymes (Carbohydrate Active enZymes) annotations were obtained through the CAZyme team (www.cazy.org), and the data were integrated with PRINGO (Miyauchi et al., 2020; Plett et al., 2023). Transposable element (TE) identification was performed with RepeatModeler and RepeatMasker (Smit and Hubley, 2015a; Smit et al., 2015b) and processed using TINGO (Morin et al., 2019). The transcript read counts were normalized and calculated differential expression of genes (DEGs) with DESeq2 (Love et al., 2014), and genes with statistically significant differences in expression were identified (FDR adjusted p < 0.05). The quality control of the transcripts was performed and the output of DEGs was integrated using SHIN+GO (Jurak et al., 2018; Ruytinx et al., 2021). The output files obtained above and functional annotations from JGI Mycocosm were integrated and visualised with VINGO (Looney et al., 2022). Several omics data sets were combined to investigate the expression profile of the BGCs in *O. maius* in symbiotic conditions and when exposed to cadmium: 1) genomic coordinates of genes and functional annotations are available on the JGI website 2) RNA-seq data are available on NCBI (Accession ID: GSE63922). Global up and down-regulation of the gene clusters was determined by considering as “regulated” a cluster where at least 50% of the genes were either up or down-regulated (i.e. ratio of 0.5 = number of up/down regulated genes/total gene number per gene cluster).

### Phylogenetic analysis of *O. maius* PKS genes and PKS domain description

Genes putatively coding for secondary metabolites in *O. maius* were previously identified by Martino et al. (2018). The 45 predicted PKSs identified in the *O. maius* genome, together with representative fungal PKSs retrieved from the literature, were selected for phylogenetic analysis. Protein sequences were analyzed using the NCBI Batch Web CD-Search Tool to identify conserved catalytic domains. In particular, ketosynthase (KS) domains were identified and extracted for subsequent phylogenetic reconstruction using the University of Maryland PKS/NRPS Analysis Web Server. The amino acid sequences of the KS domains from the 45 *O. maius* PKSs were aligned using MAFFT v7.475 (Katoh et al., 2019) with the E-INS-i iterative refinement strategy (Katoh et al., 2002). Sequence alignments were inspected and edited in BioEdit 7.0.9 (Hall, 1999). Poorly aligned positions and highly divergent regions were removed using Gblocks 0.91b (Castresana, 2000) in order to improve alignment quality prior to phylogenetic analysis. The KS alignments were then processed by IQ-TREE software (v2.1.3) (Trifinopoulos et al., 2016) to obtain phylogenetic trees using the default parameters (combine ModelFinder, tree search, ultrafast bootstrap, and SH-aLRT test). Maximum likelihood (ML) phylogenetic reconstruction was performed according to the method of Guindon and Gascuel (2003) through the PhyML program implemented in the Phylogeny.fr platform in advanced mode (Dereeper et al., 2008). The phylogenetic tree was reconstructed using the maximum likelihood method implemented in the PhyML program (v3.1/3.0 aLRT). The phylogenetic tree was visualized and annotated using Interactive Tree Of Life (iTOL; Letunic and Bork, 2024). Tree data import, manipulation, and graphical representation were performed in R using the packages *treeio, ggtree, aplot* and *gggenes*. Reference PKS sequences associated with experimentally characterized metabolites were retrieved from the MIBiG (Minimal Information on a Biosynthetic Gene Cluster) database (Medema et al., 2015; Zdouc et al., 2025).

### Expression profiles of polyketide synthase genes in *O. maius*

The transcription pattern of the *O. maius* PKS genes in symbiosis and upon cadmium exposure was also investigated. Normalised expression values and differential gene expression were extracted from previously published RNA-seq data (NCBI - GEO accession: GSE63922 and GSE119266). In brief, transcript raw read counts from biological replicates of mycorrhizal root samples were normalised and used to calculate log2 fold changes compared to the free-living mycelia using DESeq2 (Love et al., 2014). Normalised reads were consistent and comparable between all conditions tested (**Supplementary Figure 1**). Genes showing either very low raw reads or adjusted p-value (FDR) > 0.05 were excluded. A hierarchical clustering analysis of symbiosis regulated (FDR p-value<0.05) *O. maius* PKS coding genes was performed, by merging the expression level data as normalized log2 reads and protein IDs for *O. maius* PKSs using the R package Pheatmap (Kolde, 2010).

### Genetic transformation and characterization of *O. maius* PKS knockout mutants

#### Construction of the pCAMBIA0380-Hyg-PKS-KO- disruption vector and *Agrobacterium tumefaciens*-mediated transformation

The *OmPKS197601* gene was identified as a suitable candidate for gene knockout. *OmPKS197601*-null mutants were obtained through *Agrobacterium tumefaciens*-mediated (ATM) homologous recombination according to the protocol reported in Abbà et al. (2009). The vector was obtained by using the In-Fusion HD Cloning Kit (Takara Bio) according to the manufacturer’s instructions. First, the vector pCAMBIA0380 was linearized by digestion with *Bgl*II and *Sma*I restriction enzymes in two consecutive rounds, followed by gel-elution (Wizard-R-SV gel and PCR clean-up system PROMEGA). The three fragments to be cloned into the vector were obtained using the CloneAmp HiFi PCR premix (Takara Bio): the *hph* has been PCR amplified from the pCAMBIA0380-hyg vector, while the 5′ upstream flanking region (1040 bp) and the 3′ downstream flanking region (1,040 bp) of the PKS have been amplified from *O. maius* genomic DNA (**Supplementary Figure 2**). Amplicons were then purified from gel. The linearized plasmid and the three amplicons have been mixed in 10 μl at 1:1:2:2=backbone:hph:5’:3’ ratio, the mix was incubated 15 min at 50 °C and cloned in Stellar Competent Cells (Takara Bio) according to the manufacturer suggestions. The vector sequence was checked by PCR and Sanger sequencing. The vector (1.2 μg) was cloned into *Agrobacterium tumefaciens* LBA1100, the obtained clones were checked for transformation by PCR, and one was used to transform ungerminated *O. maius* conidia according to the protocol described in Abbà et al. (2009).

### Identification of *OmPKS197601*-null mutants by PCR and Southern Blot

Fungal transformants were screened by PCR. A small portion of each fungal colony was collected and boiled for 15 min in 20 μl of 10 mM Tris HCl pH 8.2, vortexed for 1 min and centrifuged 15 min at room temperature. Then, 2 μl of the supernatant were used directly for PCR amplification without any further purification, to check for the deletion of *OmPKS197601* and for the insertion of the *hph* gene. The putative *OmPKS197601*-null mutants identified were further validated by PCR using one primer designed on the genome at the 5′ and 3′ of the homologous recombination site, and one primer designed on the *hph* gene (respectively 5PKS-f1/Hyg3f and 3PKS-r1/Hyg6r). All the primers used are reported in **Supplementary Table 1**. The positive *OmPKS197601*-null mutants were further analyzed through Southern blot hybridization to verify single-copy integration of the disruption cassette in the genome. Eleven micrograms of genomic DNA from the deletion mutants and from the wild-type were digested overnight with *Bg*lII (PROMEGA) at 37 °C and size-fractionated on 1% (w:v) agarose TAE 1X gel. The separated restriction fragments were blotted onto a nylon membrane following standard procedures (Di Vietro et al., 2014). Hybridization with a probe designed on the hph cassette was performed and detected with a chemiluminescent detection system (ECL direct DNA labelling and detection system; GE Healthcare, UK) according to the manufacturer’s recommendations.

### Growth tests on zinc and cadmium added culture media

Growth of wild type *O. maius* and *OmPKS197601*-null mutants was tested in the presence of metal compounds. In particular, the Czapek-dox medium was supplemented with 0.15 mM or 0.3 mM Cd (as 3CdSO_4_٠8H_2_O), 10 mM or 15 mM Zn (as ZnSO_4_٠7H_2_O). Prior to fungal inoculation, sterile cellophane membranes were placed on the agar surface to provide a convenient means of removing the mycelium from the plate. The membranes were first boiled for 15 min in 10 mM EDTA (disodium salt, dihydrate, SIGMA), rinsed with distilled water and then autoclaved. Fungal colonies were removed after 30 days, dried overnight at 55 °C and then weighted. Data have been obtained from five or six replicates for each strain and each condition (N = 5 or 6, for a total of 105 observations). *O. maius* null-mutants were kept on selective Czapek-dox medium containing hygromycin (100 μg/ml) and cefotaxime (200 μM).

### *In vitro* mycorrhizal synthesis

Seeds of *V. myrtillus* obtained from Les Semences du Puy (Le Puy-En-Velay, France) were surface-sterilized (70% ethanol (v:v), 0.2% Tween 20 for 3 min; rinsed twice with sterile water, soaked in 0.25% sodium hypochlorite for 15 min, rinsed three times with sterile water) and germinated on 1% (w/v) water agar in the dark for 2 weeks. Axenic *V. myrtillus* seedlings were then moved for 1 month to a growth chamber (16-h photoperiod, light at 170 μmol m^−2^ s^−1^, temperature at 23 °C day and 21 °C night). *In vitro* mycorrhizal resynthesis trial with wild type *O. maius* and *OmPKS197601*-null mutants was performed in Petri plates containing the Modified Melin-Norkrans (MMN) medium (KH_2_PO_4_ 0.5 gL^−1^, Bovine Serum Albumin (BSA) 0.1 gL^−1^, CaCl_2_٠2H_2_O 0.066 gL^−1^, NaCl 0.025 gL^−1^, MgSO_4_٠7H_2_O 0.15 gL^−1^, thiamine-HCl 0.1 gL^−1^, FeCl_3_٠6H_2_O 0.001 gL^−1^, agar 10 gL^−1^, final pH 4.7). Seven plates were prepared for each fungal strain, the wild type and three null-mutants. A suspension of *O. maius* conidia prepared in sterile deionized water was distributed in the bottom half of the MMN Petri plates. In each plate, 8 germinated *V. myrtillus* seedlings were placed above the plated conidia suspension. The plates were sealed and kept in the growth chamber for 2 months. After this period, the *V. myrtillus* plants were sampled, stems and leaves were frozen while the roots were stained (0.1% methyl blue in 80% lactic acid overnight, destained in 25% lactic acid and stored in 85% lactic acid) and observed at the light microscope to evaluate the fungal colonization rate. The magnified intersections method (Villarreal-Ruiz et al., 2004) was adapted to quantify the percentage of fungal colonization of *V. myrtillus* hair roots as reported in Casarrubia et al. (2018). N = 16 hair roots have been observed per each strain.

### Antagonism assays

Antagonism assays were performed using *O. maius* wild-type, or *OmPKS197601*-null mutants, and 4 selected saprotrophic or pathogenic fungi: *Heterobasidion annosum* (Olson et al., 2012), *Kuehneromyces mutabilis* (Kuo, 2017), *Pythium* sp., (Kamoun et al., 2015), *Stereum hirsutum* (Floudas et al., 2012) (**Supplementary Table 2**). Each assay was performed on Czapek-dox agar medium (pH 6, Duchefa Biochemie) plates, by preparing 5 replicates for each combination tested. Selected saprotrophs/pathogenic fungi were added in the assay plates two weeks after the inoculation with the *O. maius* wild-type or *OmPKS197601*-null mutants. After 15 days of co-culture, the plates were photographed and fungal colony areas were measured by using the ImageJ software, each fungal area was measured 3 times and an average area value was calculated.

### Statistical analysis

All statistical analyses were performed in R. Data were first explored with descriptive statistics. Normality was evaluated using the Shapiro-Wilk test and, given the sample size (n > 70), confirmed with the Lilliefors-corrected Kolmogorov-Smirnov test; homogeneity of variances was assessed using an ANOVA on the absolute residuals of the model (Levene-type test). The non-parametric Kruskal-Wallis test was used to assess overall differences among strains. For the growth assays strain comparisons were then performed separately within each condition: for each of the five conditions, a Kruskal-Wallis test was applied to compare dry weight among the four strains, followed by pairwise Wilcoxon rank-sum tests between strains, with p-values adjusted for multiple comparisons using the Benjamini-Hochberg (BH) procedure. The same tests have been applied to compare the root mycorrhization by the four strains. Results were visualized as box plots with individual data points overlaid (jitter) and group means indicated. Adjusted p-values from the pairwise Wilcoxon tests were annotated directly on the plots. For antagonism assays statistical analysis of data was performed by applying the univariate ANOVA (p<0.05) test, followed by Tukey’s as posteriori test.

## Results

### Prediction of biosynthetic gene clusters in the *O. maius* genome

Genes involved in fungal secondary metabolite biosynthesis are typically organized into co-localized Biosynthetic Gene Clusters (BGCs) composed of up to 20 functionally related and often co-regulated genes. Using antiSMASH, which is based on the sequence homology search, a total of 59 BGCs potentially involved in secondary metabolite biosynthesis were predicted across 28 of the 100 scaffolds constituting the *O. maius* genome (**Supplementary Table 3**). The predicted BGCs included 25 Type I PKSs, 11 Type I PKS–NRPS hybrids, 3 indole–Type I PKS clusters, 1 Type III PKS–Type I PKS cluster, 4 NRPS clusters, 4 terpene clusters, 1 siderophore cluster, and 10 additional clusters classified as “other”. Raw antiSMASH output files are available in the **Supplementary Material** (**Folder S1**). The antiSMASH analysis also provided functional annotations of predicted biosynthetic gene clusters based on similarities with experimentally characterized clusters deposited in the MIBIG database (**Supplementary Tables 4**, **S5a, b**, **S6**).

### Expression profile of biosynthetic gene clusters in symbiosis with/without cadmium

The transcriptional regulation of the predicted BGCs was investigated in symbiosis by comparing mycorrhizal tissues with free-living mycelium. Given the high metal tolerance of the *O. maius* strain used in this work and the established role of secondary metabolites in fungal responses to environmental stress (Eagan and Keller, 2025), transcriptional regulation of the predicted BGCs in symbiosis was also investigated under cadmium exposure. To this aim, previously published RNA-seq datasets obtained from symbiotic (https://www.ncbi.nlm.nih.gov/geo/query/acc.cgi?acc=GSE63922) and cadmium-treated conditions (Casarrubia et al., 2020) were integrated with the antiSMASH predictions. Gene clusters were considered to be co-regulated when at least half of the genes within a cluster showed coordinated up- or down-regulation relative to the total number of genes composing that cluster (see Materials and Methods). When compared with the free-living mycelium, analysis of the RNA-seq dataset identified 12 co-upregulated BGCs in mycorrhizal tissues (**Supplementary Table 5a**), including two clusters (52 and 56) that were also upregulated in mycorrhizal roots exposed to cadmium treatment. Two BGCs (4 and 54) were upregulated by cadmium irrespective of the symbiotic condition. Only two BGCs were co-downregulated. Transcriptional regulation of BGCs can be visualised for scaffolds 1 and 3 in **Figure 1** (RNA-Seq data from Casarrubia et al., 2020) and in **Supplementary Figure 3** (RNA-Seq data from Martino et al., 2018). Transcriptional regulation of BGCs in the other scaffolds can be found in **Supplementary Table 8**.

**Figure 1:**
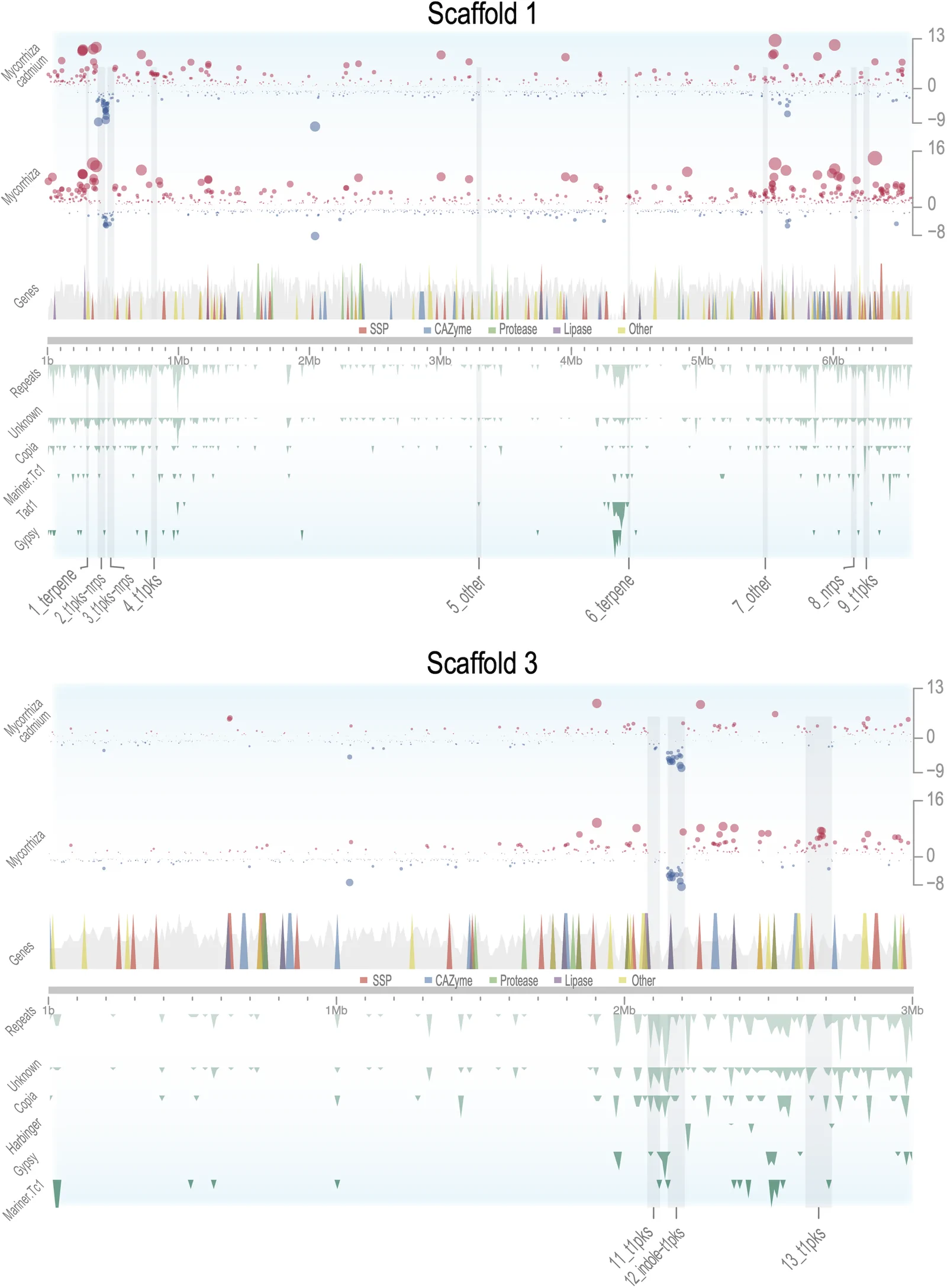
Integrated multi-omics landscape of entire scaffolds. Scaffolds 1 and 3 are presented. Gene clusters are depicted by gray vertical bars. Top panel: Up- and down-regulation of genes. The size of circles represents differential transcription levels in log2 (RNA-Seq data; Casarrubia et al., 2020). Middle panel: Density of all genes (gray) and genes encoding secreted proteins in color located in the scaffold (gray bar). Bottom panel: Density of total and individual transposable element families. See details in **Supplementary Folder S2** and **Supplementary Tables 10** and **S11**.

A more global overview of the genome organization and transcriptional regulation of predicted gene clusters also included transposable elements (TEs), and predicted secretome (i.e. CAZymes, proteases, lipases, others) (**Figures 1**; **Supplementary Figure 3**). Detailed results are in **Supplementary Table 9**.

### Phylogenetic classification and domain organization of PKSs

Out of the 59 BGCs predicted in the *O. maius* genome, 39 BGCs included one or more PKS genes, belonging to different types (**Supplementary Table 4**). To characterize further these proteins, the complete *O. maius* genome was examined and revealed 45 PKS coding genes (**Supplementary Table 6**). The domain organization of the predicted PKS proteins was analyzed to infer their potential biosynthetic functions and structural classes. PKS enzymes contain combinations of catalytic domains responsible for chain elongation and reductive modifications, and domain composition can provide insights into the nature of the predicted metabolites. Ketosynthase (KS) domains were extracted from all predicted PKSs and from representative, functionally characterized fungal PKSs retrieved from the literature and from the MIBiG database. Phylogenetic analysis were subsequently performed using Maximum Likelihood and Neighbor-Joining approaches. The phylogenetic reconstruction (**Figure 2**) grouped the *O. maius* PKSs into distinct clades. A large clade including 23 *O. maius* PKSs was characterized by the presence of an enoylreductase (ER), consistent with a highly reducing iterative type I PKS. The presence of ketoreductase (KR), dehydratase (DH), and enoylreductase domains suggests that enzymes in this clade belong to the highly reducing PKS (HR-PKS) class. Identified polyketides produced by reference fungal PKSs in this clade included lovastatin-type metabolites, fumonisins and reduced toxins. Another large clade included 18 *O. maius* PKS proteins, exhibiting a domain organization typical of non-reducing PKSs (NR-PKSs) and comprising starter-unit ACP transacetylase (SAT), keto synthase (KS), acyltransferase (AT), and acyl carrier protein (ACP). Proteins in this clade lacked reductive domains such as KR, DH, and ER. Non-reducing PKSs typically synthesize aromatic polyketides, and identified polyketides produced by reference fungal PKSs in this clade included melanins, antibiotics or toxin metabolites. The product template (PT) domain was not identified as the antiSMASH version used predates PT domain detection (Blin et al., 2019). A small clade including 4 *O. maius* PKSs displays the domain architecture consistent with partially reducing polyketide synthases (PR-PKSs), comprising KS, AT, DH, KR and ACP domains. These PKSs are typically involved in the biosynthesis of partially reduced fungal polyketides. These data suggest functional diversification of PKS pathways in this ericoid mycorrhizal species.

**Figure 2:**
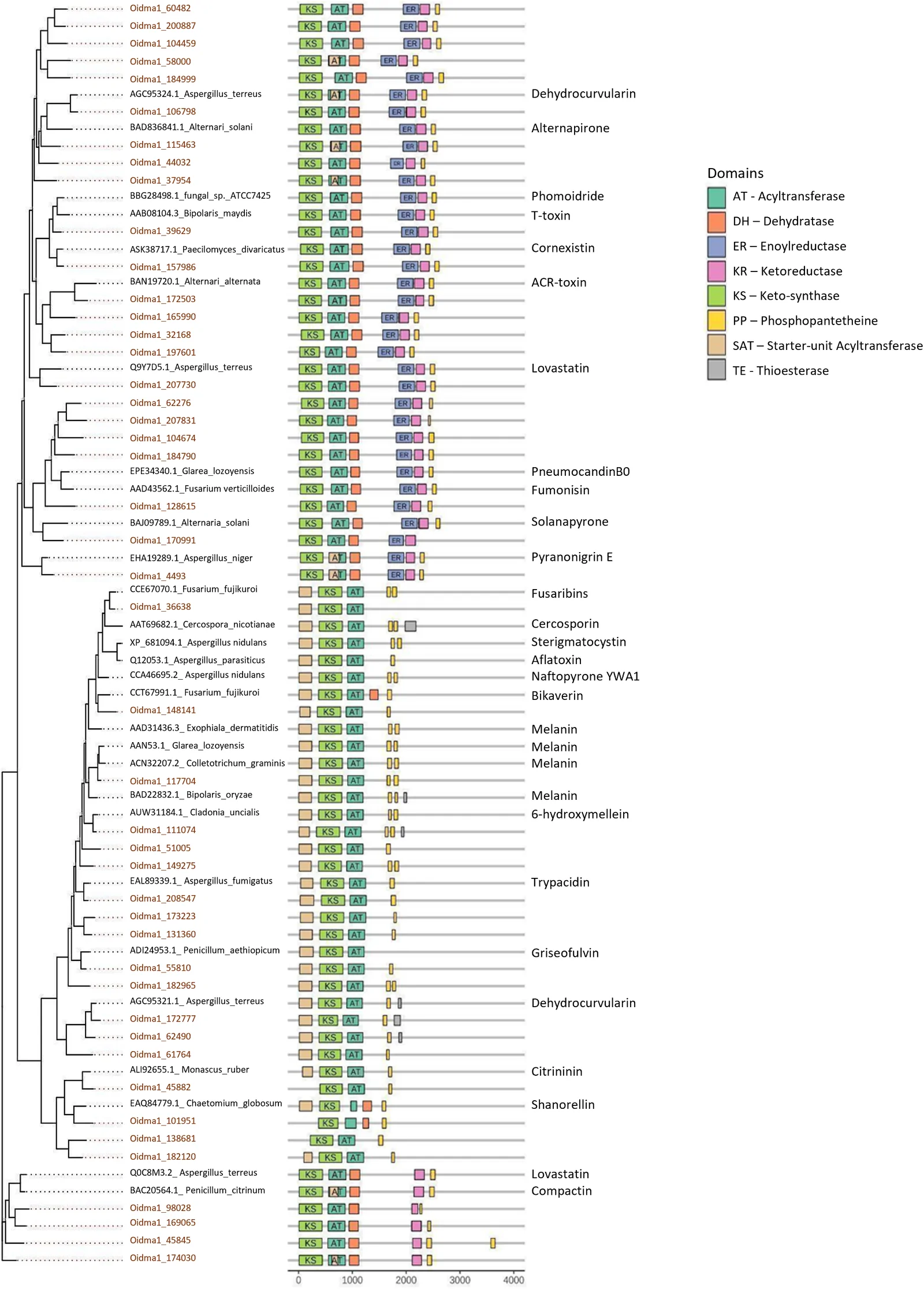
Neighbor-joining phylogenetic tree of *O. maius* and well-characterized PKS sequences from other species. Genealogy of PKSs and PKS-NRPSs was inferred by N-J analysis (MEGA 10.0.5) of the aligned amino acid sequences of the KS domains. Products of each well-characterized PKS enzyme are indicated on the tree. Branch length indicates the number of inferred amino acid changes. Only branch nodes with >50% support are shown. PKS and PKS-NRPS domains from *O. maius* were annotated using the anti-SMASH 5.0 tools.

### Differential expression of PKS genes during symbiosis

The expression profiles of *O. maius* PKS genes were analyzed using normalized log2-transformed RNA-seq read counts from previously published RNA-seq datasets obtained from symbiotic (https://www.ncbi.nlm.nih.gov/geo/query/acc.cgi?acc=GSE63922) and cadmium-treated conditions (Casarrubia et al., 2020). Hierarchical clustering analysis revealed distinct transcriptional patterns associated with different fungal PKS genes in the mycorrhizal symbiosis *vs* the free-living condition (**Figure 3**; **Supplementary Table 7**). The genes with IDs 39626 and 32168 were excluded from visualization because of insufficient transcript read coverage. Among the remaining 43 predicted PKS-coding genes, a group of 16 PKS genes was not (or lowly) expressed in both conditions (mycorrhizal symbiosis *vs* free-living mycelium), whereas a group of 5 PKS genes was not (or lowly) expressed in the Free-living mycelium but upregulated in symbiosis (**Figure 3**). Two PKS genes (IDs 101951 and 174030; highlighted in **Figure 3**) were highly expressed in the free-living mycelium but strongly downregulated under symbiotic conditions. The remaining PKS genes displayed less strongly regulated expression patterns.

**Figure 3:**
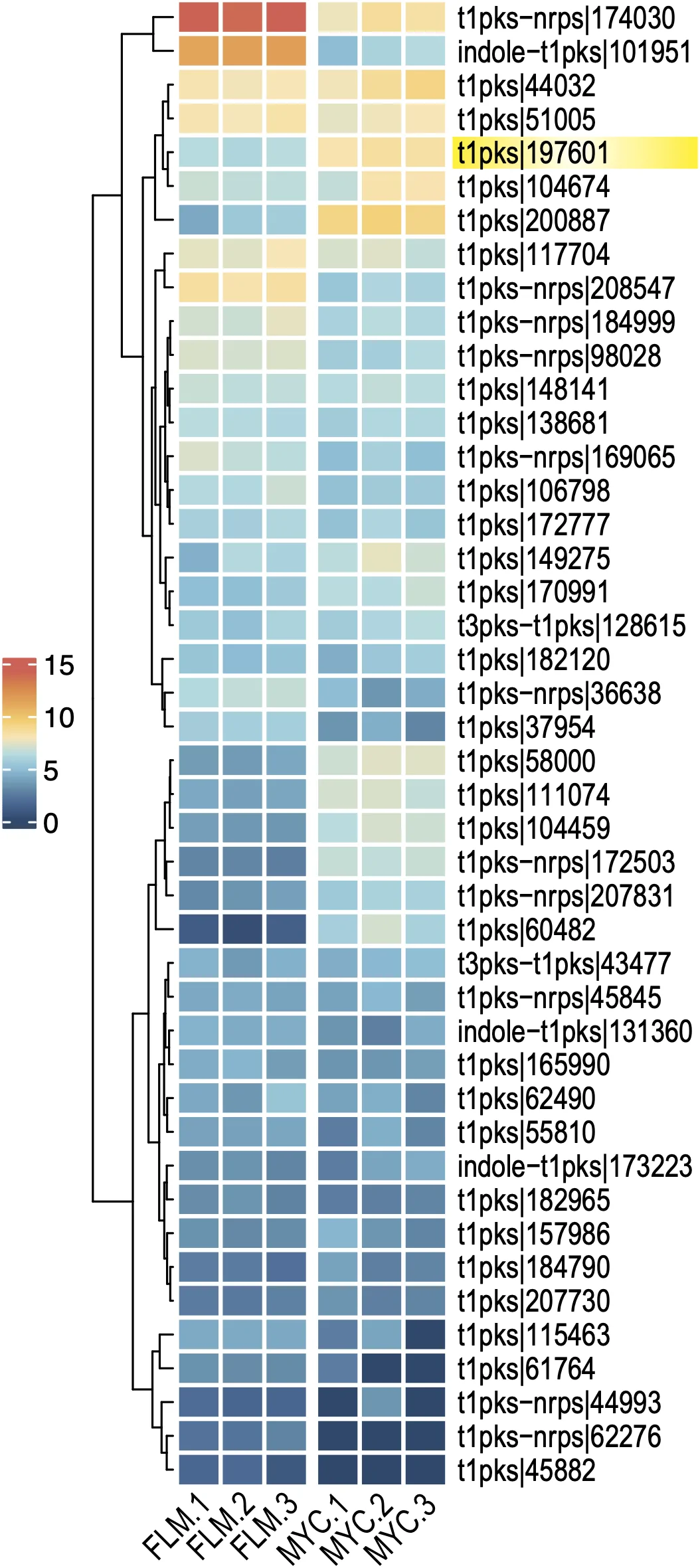
Hierarchical clustering analysis of *O. maius* PKS coding genes regulated in symbiosis (p<0.05). The expression level is shown as normalized log2 reads. PKS type and protein IDs are reported on the right side of the image. MYC: Mycorrhizal samples. FLM, Free living mycelium. The gene ID 197601, chosen for genetic transformation, is highlighted in yellow (RNA-Seq data from Casarrubia et al., 2020).

Polyketide synthases (PKSs) are strongly implicated in fungal responses to environmental stress, although their role is often indirect through the production of stress-protective secondary metabolites. Therefore, PKS gene expression in mycorrhizal and free-living conditions was also compared in the presence of cadmium (**Supplementary Table 7**). With or without cadmium, most PKS genes maintained the same expression profile in symbiosis, apart from a few exceptions. In particular, in the absence of cadmium, PKS genes with IDs 104459 and 149275 were significantly upregulated in symbiosis, but they were not regulated when exposed to this heavy metal. Cadmium also significantly downregulated the expression of PKS genes with IDs 104674 and 44032 in symbiosis (**Supplementary Table 7**). The PKS gene with ID 197601 (*OmPKS197601*) was significantly upregulated in the mycorrhizal condition, both with and without Cd (**Supplementary Table 7**). *OmPKS197601* belongs to the large clade of putative highly reducing PKS (HR-PKS) (**Figure 2**) and it was chosen for further characterization mainly based on its transcriptomic expression pattern (see next paragraph).

### Generation and molecular validation of *OmPKS197601* knockout mutants

The PKS encoded by *OmPKS197601* (highlighted in **Figure 3**) was selected to investigate the biological role of PKSs in free-living mycelium and during symbiosis through targeted gene knockout via *Agrobacterium tumefaciens*-mediated homologous recombination. *OmPKS197601* was selected because it was expressed in the free-living mycelium, enabling phenotypic characterization of the null mutants under different experimental conditions, and was up-regulated under symbiotic conditions, suggesting a putative role in plant-fungal interactions.

The disruption construct was obtained by modifying the pCAMBIA0380-HYG vector to replace the target gene with a hygromycin resistance cassette (**Supplementary Figure 2**). Following fungal transformation and selection on hygromycin-containing medium, 628 *O. maius* transformants were screened by PCR using primers targeting the deleted PKS region and the hygromycin resistance cassette. Three independent knockout mutants, designated M1, M2, and M17, lacked amplification of the target PKS fragment, confirming deletion of *OmPKS197601* (**Figure 4A**). Amplification of the hygromycin resistance (*hph*) cassette further confirmed successful insertion of the disruption construct (**Figure 4B**). Additional PCR analysis using primers designed on genomic regions flanking the recombination site and within the hygromycin cassette (supposed to have replaced the selected PKS) demonstrated the correct integration of the disruption construct at the expected genomic locus (**Figures 4C, D**). Because *A. tumefaciens*-mediated transformation may occasionally result in multiple ectopic integrations, Southern blot analyses were performed using a probe targeting the hygromycin resistance cassette. The three selected mutants showed a single insertion event within the genome (**Figure 4E**). The relatively low homologous recombination frequency obtained in this study (approximately 0.5%) may be related to the large size of the target PKS gene, which spans approximately 12 kb.

**Figure 4:**
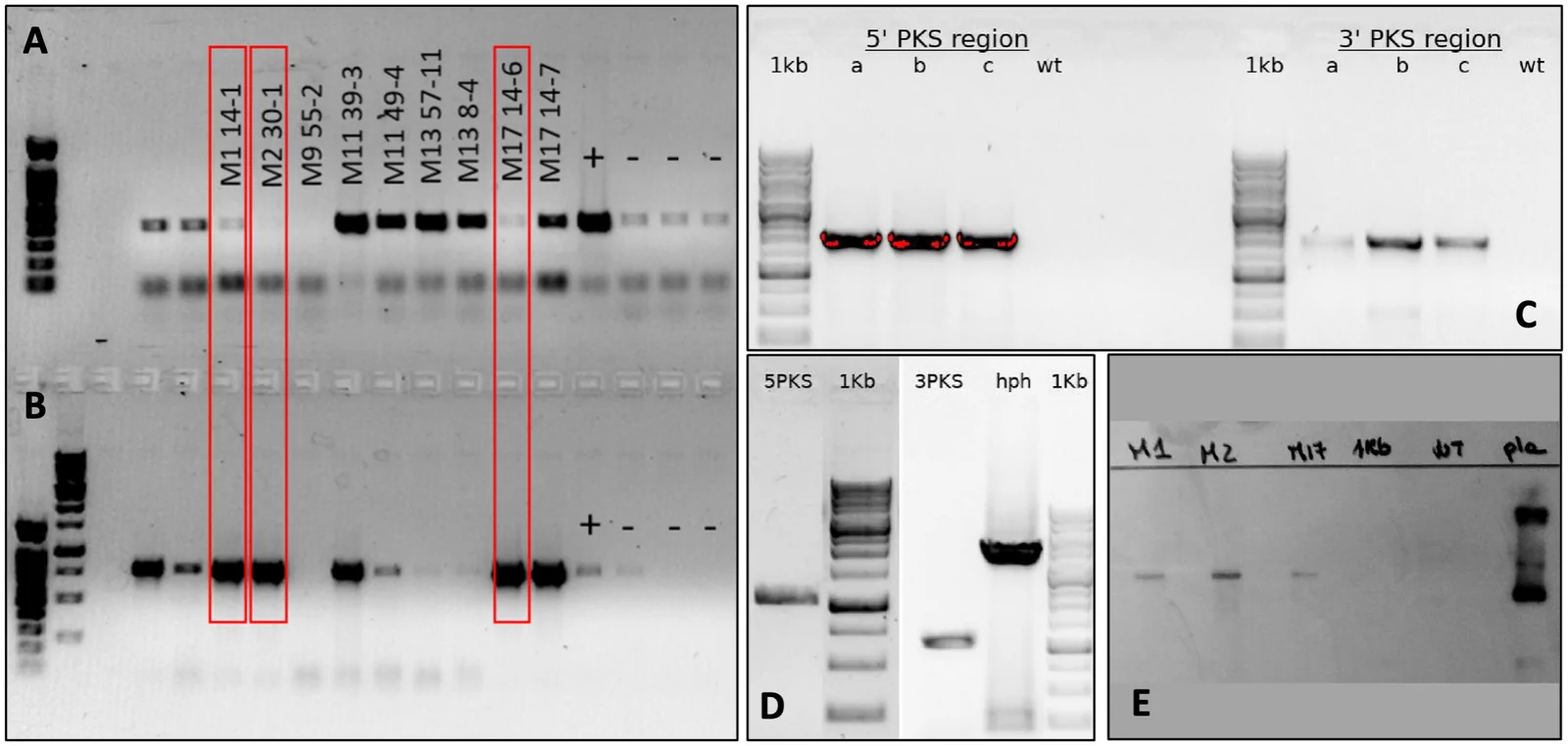
PCR-screening of putative *O. maius* PKS null-mutants. **(A)** PKS-r2/PKS-f2 primers designed on the AT domain of *OmPKS197601*; **(B)** Hyg2r/Hyg4f primers designed on the hygromycin resistance gene cassette. **(C)** Amplification of the flanking regions of the mutated site with a primer designed on the resistance cassette and a primer on a genomic region flanking the deleted gene, upstream (5’) and downstream (3’); a: M1; b: M2; c: M17; WT: wild-type. **(D)** PCR amplification of the 5’ and 3’ PKS-flanking sequences and of the hph-cassette. **(E)** Southern blot analysis to check the number of hph-cassette insertions in the *OmPKS197601*-null mutants: a single insertion was detected in the 3 selected mutants M1, M2 and M17. WT, wild-type (negative control); pla, disruption vector (positive control). 1Kb: bp-size ladder.

### Phenotypic characterization of knockout mutants

The *OmPKS197601*-null mutants were characterized for their ability to establish mycorrhizal symbiosis, tolerate heavy metals, and antagonize the growth of other fungi. The ability of the mutants to establish ericoid mycorrhizal associations with *V. myrtillus* seedlings was evaluated *in vitro*. Microscopic observations and quantitative analysis of root colonization revealed no statistically significant differences between the wild-type *O. maius* strain and the three knockout mutants (**Supplementary Figure 4**). Although mutant M2 displayed a tendency toward reduced intracellular coil formation compared with the wild-type strain, this difference was not statistically significant.

Given the high heavy metal tolerance of *O. maius* strain Zn (Martino et al., 2007), growth of the wild-type strain and the three knockout mutants was also evaluated on culture media supplemented with cadmium or zinc (**Figure 5**). No significant differences in fungal biomass production or metal tolerance were detected between the wild-type and mutant strains under any of the tested conditions. The absence of detectable phenotypic differences suggests that *OmPKS197601* is not essential for heavy metal tolerance under the experimental conditions tested, although compensatory regulation by additional PKS pathways cannot be excluded.

**Figure 5:**
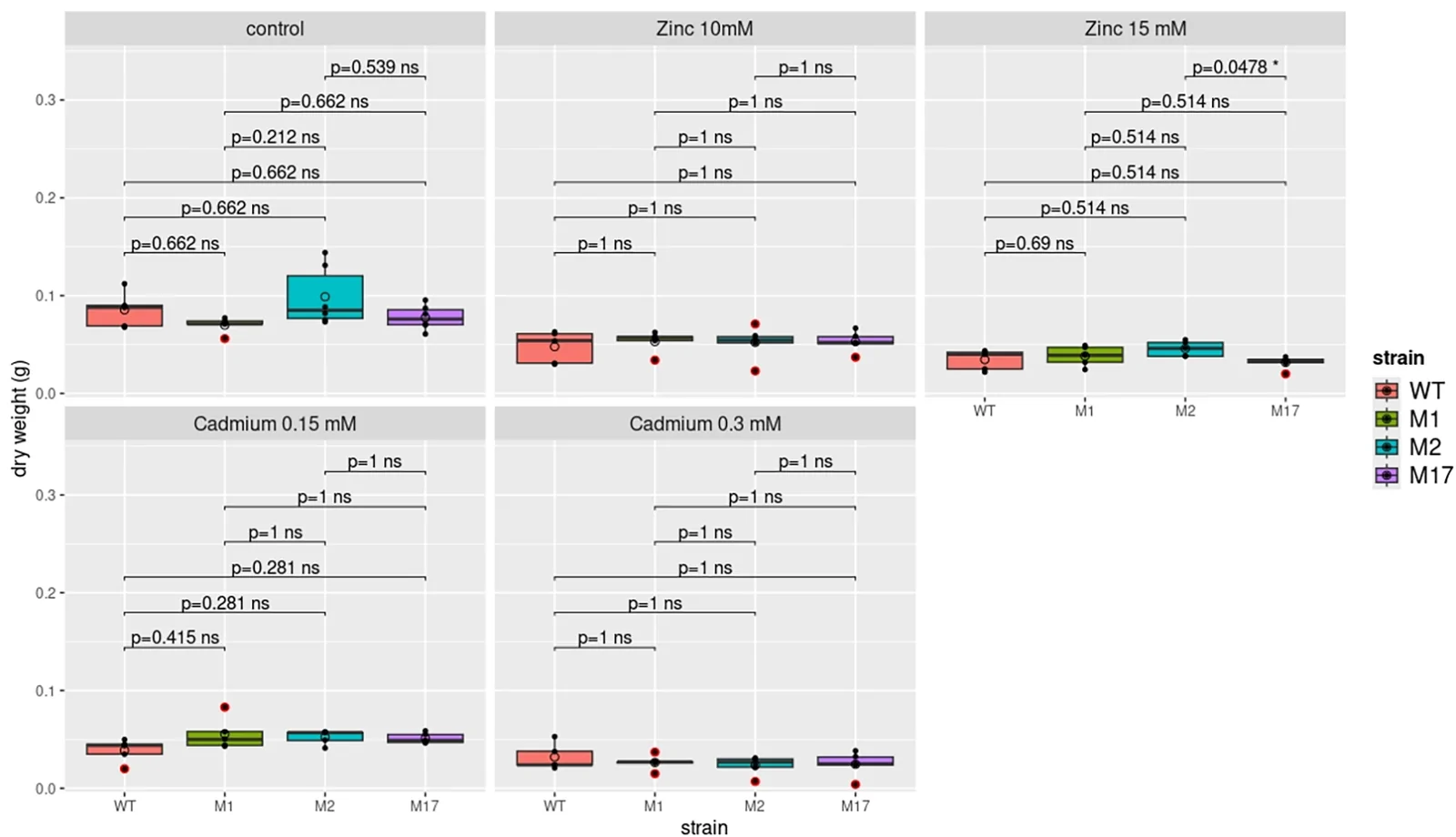
Heavy metal tolerance assays of *O. maius* wild type and of the three *OmPKS197601*-null mutants. The line that divides the box represents the median of the data, the empty round point represents the mean, the small black points represent the single data, the points with red line represent the outliers, the whiskers represent the highest and lowest values, excluding outliers. Adjusted p-values from the pairwise Wilcoxon tests are reported. ns= not significant.

In contrast, significant differences were observed during dual-culture confrontation assays performed with selected plant pathogenic and saprotrophic fungi. The fungal species selected for these experiments — *H. annosum*, *K. mutabilis*, *Pythium* sp., and *S. hirsutum* (**Supplementary Table 2**) — are representative of fungi colonizing peatland ecosystems (Thormann and Rice, 2007). The antagonism assay was initially performed with the three *OmPKS197601* mutants, but results are shown only for mutant M2 because of the most stable and clear phenotype. In all confrontation assays (**Figure 6**), the growth area of the tested fungi was significantly smaller (p<0.05) when co-cultivated with the *O. maius* wild-type strain compared with plates containing the knockout mutant M2. This pattern was consistently observed for *H. annosum*, a plant pathogenic fungus for which literature data already indicated antagonistic activity by *O. maius*; for *K. mutabilis*, a saprotrophic fungus that colonizes peat bog environments; for *Pythium* sp., a genus that includes saprotrophic fungi, but also plant pathogenic fungi; for *S. hirsutum*, a saprotrophic fungus that colonizes peat bog environments (**Figure 6**). These results suggest that the PKS-derived metabolite likely associated with *OmPKS197601* may contribute directly or indirectly to antagonistic fungal interactions and may participate in ecological competition within peatland and rhizosphere environments.

**Figure 6:**
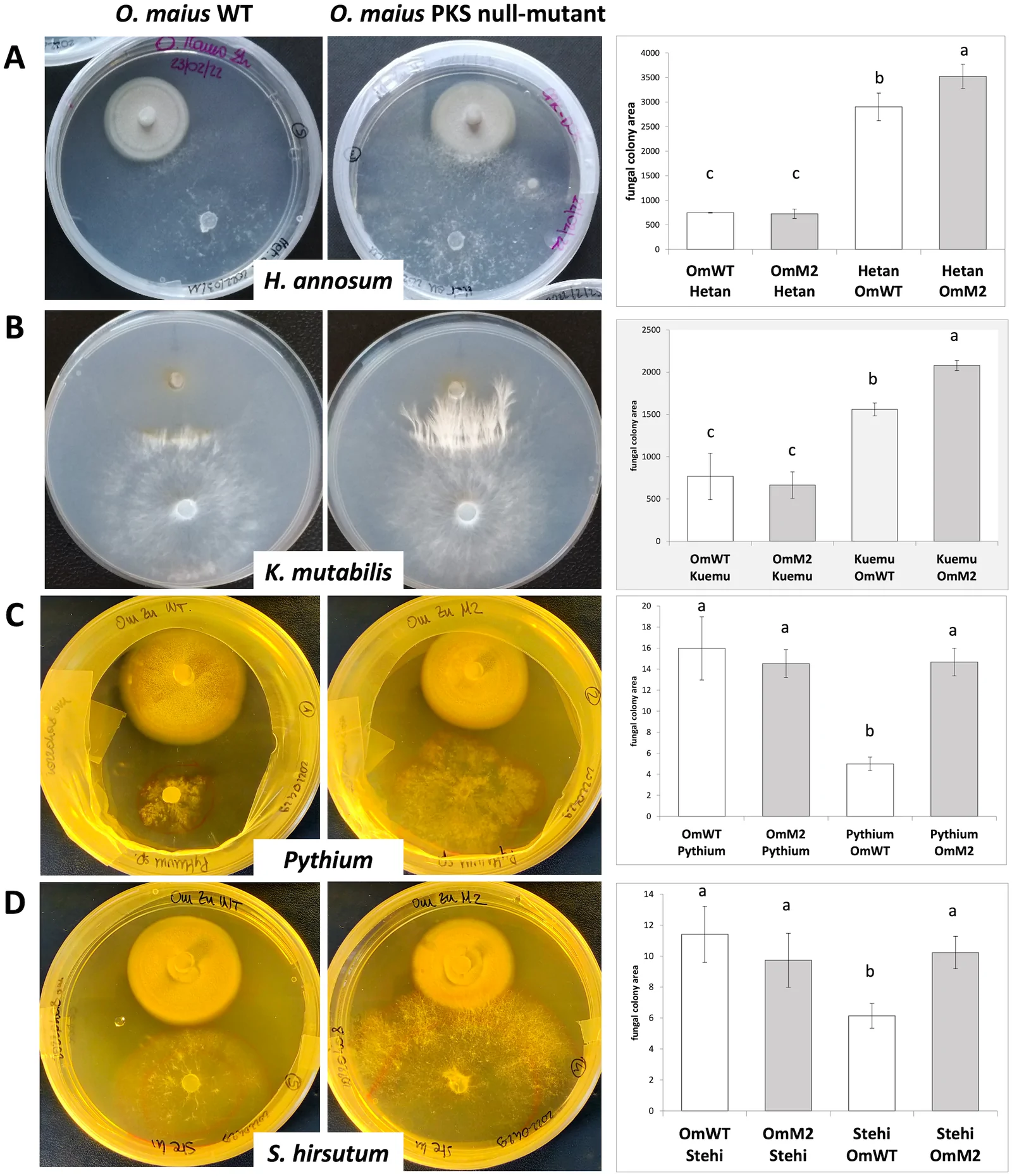
Antagonism assays. *O. maius* WT and *O. maius* PKS null-mutants’ confrontation cultures (n=5) with **(A)** *Heterobasidion annosum*, **(B)** *Kuehneromyces mutabilis*, **(C)** *Pythium* sp., **(D)** *Stereum hirsutum*. The histograms correspond to the area of the fungal colonies measured in the confrontation plates, the strain measured area is the first one in the histogram legend, different letters indicate samples with statistically significant differences (p<0.05): a significantly smaller growth area was measured when the tested fungi grew in front of the *O. maius* WT strain.

## Discussion

The present study reveals that *O. maius* Zn possesses a remarkably extensive repertoire of biosynthetic gene clusters (BGCs), highlighting the broad secondary metabolic potential in this ericoid mycorrhizal fungus. Genome mining identified 59 predicted BGCs distributed across multiple genomic scaffolds and comprising diverse biosynthetic classes, including PKSs, PKS–NRPS hybrids, terpene synthases, siderophores, and indole-associated clusters. These findings are consistent with growing evidence that mycorrhizal fungi retain complex secondary metabolic capacities despite evolutionary transitions toward symbiotic lifestyles (Kohler et al., 2015; Looney et al., 2022). In fungi, BGCs are increasingly recognized as central determinants of ecological adaptation, mediating interactions with hosts, competitors, and the surrounding microbiota through the production of specialized metabolites involved in signaling, nutrient acquisition, stress tolerance, and antagonism (Keller, 2019; Medema et al., 2015). The coordinated transcriptional regulation observed for several *O. maius* BGCs during symbiosis and cadmium exposure supports the hypothesis that secondary metabolism contributes to the adaptive responses of ericoid mycorrhizal fungi to both biotic and abiotic environmental cues.

Within this broad secondary metabolic landscape, PKS-containing clusters represented the dominant BGC category in the *O. maius* genome. Comparative genomic analysis previously indicated that *O. maius* possesses one of the largest complements of PKS genes among fungi with different ecological strategies (Martino et al., 2018). The present study extends this observation by integrating phylogenetic reconstruction, transcriptomics, and functional genetics. The abundance and diversity of PKS genes strongly suggest that secondary metabolism contributes to the ecological versatility and adaptive potential of this fungus. Fungal PKSs generate structurally diverse metabolites involved in pigmentation, oxidative stress protection, developmental regulation, antimicrobial activity, and ecological competition (Cox, 2007; Keller, 2019). The predominance of highly reducing and non-reducing PKSs identified in *O. maius* suggests the capacity to synthesize a wide spectrum of aliphatic and aromatic polyketides potentially relevant for adaptation to nutrient-poor and environmentally stressful habitats colonized by Ericaceae and their associated fungi.

Although PKSs have been extensively studied in pathogenic and biocontrol fungi, their roles in mycorrhizal systems remain comparatively poorly understood. The transcriptional activation of several *O. maius* PKS genes and BGCs during symbiosis with *V. myrtillus* suggests that secondary metabolites may contribute to the establishment or maintenance of ericoid mycorrhizal interactions. Increasing evidence indicates that specialized metabolites participate in the molecular dialog underlying fungal–plant symbioses by modulating host immunity, shaping rhizosphere microbial communities, and mediating ecological specificity (Schirawski and Perlin, 2018). Although the ecological functions of PKS-derived metabolites in ericoid mycorrhizal fungi remain largely unresolved, comparative genomic studies indicate that these fungi have retained an extensive repertoire of secondary metabolite biosynthetic genes despite their mutualistic lifestyle, suggesting that specialized metabolites continue to play important ecological roles beyond pathogenicity. In this context, PKS-derived metabolites may contribute not only to plant–fungus interactions but also to microbial competition and adaptation to the chemically and biologically complex soil environment inhabited by Ericaceae. Retention and diversification of secondary metabolism pathways have been also described in ectomycorrhizal and arbuscular mycorrhizal fungi (Venice et al., 2020; Looney et al., 2022), supporting the view that secondary metabolism remains functionally important in mutualistic fungi. Genomic analysis of arbuscular mycorrhizal fungi, including *Gigaspora margarita*, revealed the presence of complex PKS and hybrid PKS–NRPS genes with mosaic architectures (Venice et al., 2020, 2021), suggesting that these biosynthetic pathways have been evolutionarily conserved despite the obligate biotrophic lifestyle of these fungi. Similarly, ectomycorrhizal fungi retain diverse secondary metabolite biosynthetic pathways despite reductions in plant cell wall-degrading enzymes associated with the transition from saprotrophy to mutualism (Martin et al., 2016; Looney et al., 2022). Collectively, these observations indicate that the retention of PKS biosynthetic capacity is a common feature across the major mycorrhizal lineages and support the hypothesis that PKS-derived metabolites contribute to ecological fitness through functions such as chemical signaling, microbial competition, environmental adaptation, and interactions with the surrounding microbiota, even though the identity and biological activity of many of these metabolites remain to be elucidated.

The induction in *O. maius* of specific BGCs and PKS genes under cadmium exposure additionally suggests a role for secondary metabolism in environmental stress responses. Fungal secondary metabolites are known to participate in oxidative stress protection and ecological resilience (Eisenman and Casadevall, 2012; Keller, 2019), and the transcriptional responsiveness observed here is consistent with the high metal tolerance previously described for *O. maius* Zn (Daghino et al., 2016). Nevertheless, disruption of the strongly induced gene *OmPKS197601* did not significantly alter cadmium or zinc tolerance, nor the capacity to establish mycorrhizal symbiosis under the experimental conditions tested. Several explanations may account for the absence of a detectable phenotype. First, functional redundancy among the numerous PKS genes present in the *O. maius* genome may compensate for the loss of a single biosynthetic pathway. Such redundancy is a common feature of fungal secondary metabolism, where multiple biosynthetic clusters can produce structurally or functionally related metabolites (Fischbach et al., 2008). Second, the ecological roles of PKS-derived metabolites may become evident only under environmental conditions more complex than those reproduced *in vitro*. Finally, PKS products may function during specific developmental stages or ecological contexts not captured in the present experimental system. The absence of significant differences in cadmium and zinc tolerance between the wild-type strain and the knockout mutants suggests that *OmPKS197601* is not directly required for metal resistance under the tested conditions. Nevertheless, the strong transcriptional induction observed during cadmium exposure in the symbiotic condition indicates that PKS pathways may still participate in stress-responsive metabolic networks. Secondary metabolites are known to contribute to oxidative stress protection and environmental adaptation in fungi (Eisenman and Casadevall, 2012), and compensatory activation of additional PKS pathways may occur in the knockout mutants. Future metabolomic analysis will be important to identify the OmPKS197601-derived secondary metabolite(s), whereas transcriptomics and metabolomics will clarify whether alternative biosynthetic pathways are activated following disruption of *OmPKS197601*.

In contrast, knockout mutants, in particular M2, displayed a clear reduction in antagonistic activity against both pathogenic and saprotrophic fungi, indicating that the metabolite synthesized by OmPKS197601 may contribute to fungal competitive interactions. Similar ecological functions have been demonstrated for PKS-derived metabolites in other filamentous fungi, particularly in species involved in biological control. For example, PKS-derived metabolites in *Trichoderma harzianum* participate in mycoparasitism and pathogen inhibition (Baker et al., 2012; Yao et al., 2016). Interestingly, the antagonistic activity of *O. maius* was observed not only against plant pathogens such as *H. annosum* and *Pythium* sp., but also against saprotrophic fungi inhabiting similar ecological niches. These species are characteristic colonizers of peatland environments (Thormann and Rice, 2007), where microbial competition for nutrient resources is particularly intense. The antagonistic and potential biocontrol activity of *O. maius* is consistent with previous reports showing inhibitory effects of ericoid mycorrhizal fungi against root pathogens, including *Phytophthora cinnamomi* and *H. annosum* (Schild et al., 1988; Qian et al., 1998). Competitive interactions between symbiotic and saprotrophic fungi are known to influence nutrient cycling, microbial community structure, and host plant performance (Pereira et al., 2012; Baptista et al., 2021). In nutrient-poor ecosystems such as heathlands and peatlands, the production of antagonistic metabolites by *O. maius* may therefore represent an important ecological strategy and could contribute not only to niche protection and resource competition, but also indirectly to host plant performance through suppression of deleterious microorganisms in the rhizosphere.

More broadly, the ecological importance of PKS-derived secondary metabolites in symbiotic systems is increasingly recognized beyond fungal–plant interactions. For example, the phytotoxin rhizoxin, produced by bacterial endosymbionts of *Rhizopus microsporus*, is essential for rice seedling blight pathogenicity (Partida-Martinez and Hertweck, 2005), whereas bryostatins produced by bacterial symbionts of marine bryozoans function as defensive compounds against predation (Sudek et al., 2007). Together, these examples highlight how secondary metabolites frequently mediate ecological interactions within symbiotic systems. From an applied perspective, the potential biocontrol role of *O. maius* secondary metabolites is particularly relevant considering the increasing agronomic importance of Ericaceae crops, including *Vaccinium* species. Ericoid mycorrhizal fungi capable of simultaneously improving plant nutrition and suppressing pathogenic microorganisms may represent valuable tools for sustainable crop production and soil management.

## Conclusions

Overall, this study highlights the remarkable secondary metabolic potential of *O. maius* and provides evidence that PKS-derived metabolites may contribute to antagonistic fungal interactions. More broadly, our findings support the emerging view that secondary metabolites are integral components of the chemical ecology of mycorrhizal fungi rather than a residual trait inherited from saprotrophic ancestors.

The specific metabolite synthesized by OmPKS197601 remains to be chemically identified, and future studies should focus on the metabolomic characterization of the compounds synthesized by OmPKS197601 and by other PKS pathways identified in *O. maius*, in order to establish direct links between biosynthetic gene clusters and their corresponding metabolites.

Integration of comparative genomics, transcriptomics, targeted mutagenesis, and metabolomic approaches will be essential to elucidate the ecological functions of PKSs in ericoid mycorrhizal fungi.

For example, genetic complementation of PKS knockout mutants would provide definitive evidence that the observed reduction in antagonistic activity is specifically attributable to the disruption of *OmPKS197601*, and transcriptomic analysis of PKS knockout mutants could provide insights into compensatory regulatory mechanisms and pathway interactions within the fungal secondary metabolism network. The application of CRISPR/Cas9-based genome editing approaches may further facilitate the functional dissection of large multidomain PKS genes and improve gene-targeting efficiency in *O. maius*. Finally, elucidating the ecological and biocontrol functions of PKS-derived metabolites may contribute to the development of sustainable strategies for the cultivation and protection of economically important Ericaceae species, including *Vaccinium* crops.

## Data Availability

The datasets presented in this study can be found in online repositories. The names of the repository/repositories and accession number(s) can be found in the article/**Supplementary Material**. Source code for *Visual Omics Tools* (PRINGO, VINGO, TINGO) is available at github.com/ShingoMiyauchi.
